# The Mechanisms for Within-Host Influenza Virus Control Affect Model-Based Assessment and Prediction of Antiviral Treatment

**DOI:** 10.3390/v9080197

**Published:** 2017-07-26

**Authors:** Pengxing Cao, James M. McCaw

**Affiliations:** 1 School of Mathematics and Statistics, The University of Melbourne, Melbourne, Victoria 3010, Australia; pengxing.cao@unimelb.edu.au; 2 Centre for Epidemiology and Biostatistics, Melbourne School of Population and Global Health, The University of Melbourne, Melbourne, Victoria 3010, Australia; 3 Modelling and Simulation, Infection and Immunity Theme, Murdoch Childrens Research Institute, The Royal Children’s Hospital, Parkville, Victoria 3052, Australia

**Keywords:** model selection, target cell depletion, immune response, neuraminidase inhibitor, drug efficacy

## Abstract

Models of within-host influenza viral dynamics have contributed to an improved understanding of viral dynamics and antiviral effects over the past decade. Existing models can be classified into two broad types based on the mechanism of viral control: models utilising target cell depletion to limit the progress of infection and models which rely on timely activation of innate and adaptive immune responses to control the infection. In this paper, we compare how two exemplar models based on these different mechanisms behave and investigate how the mechanistic difference affects the assessment and prediction of antiviral treatment. We find that the assumed mechanism for viral control strongly influences the predicted outcomes of treatment. Furthermore, we observe that for the target cell-limited model the assumed drug efficacy strongly influences the predicted treatment outcomes. The area under the viral load curve is identified as the most reliable predictor of drug efficacy, and is robust to model selection. Moreover, with support from previous clinical studies, we suggest that the target cell-limited model is more suitable for modelling in vitro assays or infection in some immunocompromised/immunosuppressed patients while the immune response model is preferred for predicting the infection/antiviral effect in immunocompetent animals/patients.

## 1. Introduction

Influenza is an infectious disease targeting a host’s respiratory system, causing high morbidity and mortality worldwide [1,2]. Infection of healthy epithelial cells in the upper respiratory tract (URT) with influenza virus leads to a rapid viral reproduction and may subsequently cause symptoms from mild cough, runny nose and sore throat to severe illness. Antiviral drugs are important to alleviate influenza symptoms at the individual level and reduce transmission at the population level [3,4]. The most widely used antiviral drugs are neuraminidase inhibitors (NAI), such as oseltamivir and zanamivir [5], which (partially) block the release of influenza virus from infected cells and in turn reduce virus spread within the URT. Upon the emergence of novel influenza strains, assessing the antiviral efficacy and impact on both reducing viral replication and controlling the spread of resistance is both urgent and important [6,7,8]. This is, however, challenging due to a limited understanding of both viral and immune system dynamics in the host and the feasibility of various endpoints proposed for clinical trials [9,10,11].

Mathematical models of within-host influenza viral dynamics provide a platform on which the effect of antiviral drugs can be investigated and predicted due to their transparent structures and biologically interpretable parameters [12,13]. Previously the function of NAIs has been modelled by a constant reduction in the rate of viral production, through which altered model kinetics were investigated [11,14,15,16,17,18,19]. Those studies (also including any mentioned later) have played an important role in advancing our understanding of antiviral effects. For example, Baccam et al. found that an influenza viral infection model with a 97% reduction in viral production rate reasonably reproduced a set of viral load data [14]; Handel et al. studied the emergence and spread of NAI resistance using a model with both NAI-sensitive and NAI-resistant pools of virus and identified significant challenges related to the quantification of resistance generation [15]; Dobrovolny et al. compared the effect of NAIs against infection in human and avian hosts and demonstrated that NAIs are efficacious for both strains [17]. Other models have incorporated both viral dynamics and plasma NAI concentration dynamics (which are referred to as the pharmacodynamic-pharmacokinetic (PK-PD) models) such that the reduction in viral production rate is dependent on the time course of plasma drug concentration [20,21]. These models are more realistic and allow for the assessment of alternative dosing strategies [20], although the detailed kinetics of drug concentration are not necessarily important for high frequency dosing regimens [21].

**Figure 1:**
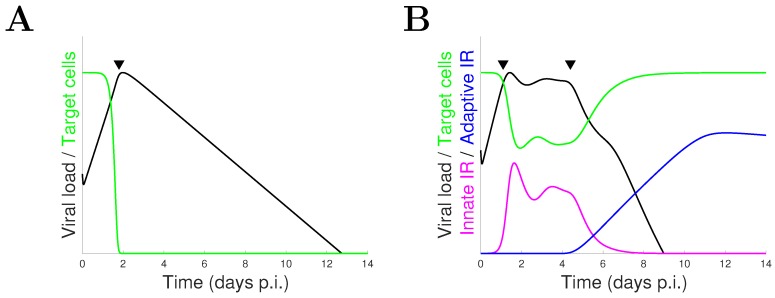
Schematic illustration of the typical solutions to the target cell-limited models (**A**) and models incorporating both innate and adaptive immune responses (**B**). For the target cell-limited models, severe depletion of target cells stops viral growth leading to viral clearance (the approximate turning point is indicated by a filled triangle). In contrast, for the models with both innate and adaptive immune responses, timely activation of the innate immune response stops viral growth and later activation of the adaptive immune response is responsible for viral clearance (the approximate times of activation are indicated by filled triangles). p.i.: post-infection.

Although influenza viral dynamic models vary from study to study, most of the models used for the assessment and prediction of NAI efficacy belong to a common model type—the target cell-limited model—which is structured to capture the essential interactions among three populations: target cells (i.e., healthy epithelial cells susceptible to virus infection), virus-infected cells and free virus. A key feature of this class of models is that target cell depletion will occur after approximately two days post-infection (p.i.) such that an effective control of viral load is primarily driven by a dramatically reduced viral production (a typical solution is given in Figure 1A). Practically, while this model may be suitable for modelling an in vitro assay [22,23] where the immune response is absent, it is less suitable for in vivo viral dynamics where viral control is thought to be dominated by various activated immune responses (IR) [24] (a schematic illustration is given in Figure 1B).

Assumed differences in the mechanism of viral control have been shown to influence the predictive power of viral dynamic models [13,15,25,26]. For the prediction of NAI effects, Handel et al. showed that a lack of NAI treatment data prevented identification of the mechanisms of NAI action and argued for the necessity to incorporate the dynamics of the immune response into models for further assessment [15]. Therefore, with the increasing attention being paid to model-based studies of antiviral efficacy and clinical applications, it is important to understand how the different mechanisms for viral control (i.e., target cell-limited viral growth versus immunity-driving viral clearance) affect model predictions of antiviral treatment outcomes.

In this paper, we first present a typical target cell-limited model and a virus dynamic model with both innate and adaptive immune responses which exhibits typical behaviour of natural infection and has been validated with multiple sources of experimental data. We analyse the model dynamics and demonstrate that the two models utilise different mechanisms to control viral load. By integrating the two virus dynamic models with a pharmacokinetic model of NAI, we then analyse the models’ behaviours and compare their predictions on how the viral load profile depends on NAI efficacy and how infection-related quantities (such as the area under the viral load curve, peak viral load, and duration of infection) are associated with NAI efficacy. Finally, we examine the predictions of the two models on treatment outcomes for different initiation times for drug administration.

## 2. Materials and Methods

In this section, we first provide details for the two models with different mechanisms of within-host viral control. The target cell-limited model which we focus on here is a system of ordinary differential equations (ODEs) with the simplest structure capturing the essential interactions among target cells (denoted by *T*), virus-infected cells (denoted by *I*) and free virus (denoted by *V*), known as the TIV model. The other model incorporates innate and adaptive immune responses and is referred to as the immune response model (IR model). The IR model is an extension of the TIV model and contains both ordinary and delay differential equations.

We then introduce a pharmacokinetic model which produces a realistic time course for plasma NAI concentration. In this study, we shall focus on oseltamivir (the most commonly used NAI) whose active metabolite, oseltamivir carboxylate (OC), plays a direct role in reducing the viral production from infected cells [5].

Finally, we introduce the infection-related statistics for both models, such as the area under the viral load curve, peak viral load, and duration of infection, which will be analysed in the Results and provide important insights into the dynamics of the two models with antiviral treatment.

### 2.1. The TIV Model

The TIV model is given by

(1) $$\begin{array}{cc} \frac{dT}{dt} & =g_{T}T\left(1-\frac{T+I}{T_{0}}\right)-\beta^{\prime }VT, \end{array}$$

(2) $$\begin{array}{cc} \frac{dI}{dt} & =\beta^{\prime }VT-\delta_{I}I, \end{array}$$

(3) $$\begin{array}{cc} \frac{dV}{dt} & =\left(1-\epsilon \right)p_{V}I-\delta_{V}V-\beta VT. \end{array}$$

Virus (quantified by viral load *V*) is produced from infected cells (*I*) at rate $p_{V}$ and is cleared at a rate $\delta_{V}$. Consumption of free virus is also via binding to target cells (*T*) at a rate βT. An NAI-induced reduction in viral production is modelled by the factor 1−ϵ where ϵ is a Hill function of OC concentration, *D*, detailed later. Target cells are replenished by logistic regrowth of the target cell pool at a basal rate $g_{T}$ but limited by the capacity $T_{0}$ and are also consumed by the invasion of free virus at a rate $\beta^{\prime }V$ (note that β and $\beta^{\prime }$ are different parameters due to different units for viral load *V* and target cells *T* [26]). Invasion of free virus into target cells produces infected cells, which are then cleared at a rate $\delta_{I}$. This model retains the fundamental structure of the majority of target cell-limited models in the literature [13].

### 2.2. The IR Model

The IR model extends the TIV model by including innate and adaptive immune response mechanisms, each of which performs a particular function over a distinct timescale [27]. The model is formulated as a system of ordinary and delay differential equations. We adopt one of our published models [27] which incorporates a comprehensive set of major immune responses and which has been validated against a number of sources of experimental data:

(4) $$\begin{array}{cc} \frac{dV}{dt} & =\left(1-\epsilon \right)p_{V}I-\delta_{V}V-\kappa_{S}VA_{S}-\kappa_{L}VA_{L}-\beta VT, \end{array}$$

(5) $$\begin{array}{cc} \frac{dT}{dt} & =g_{T}\left(T+R\right)\left(1-\frac{T+R+I}{T_{0}}\right)-\beta^{\prime }VT+\rho R-\phi FT, \end{array}$$

(6) $$\begin{array}{cc} \frac{dI}{dt} & =\beta^{\prime }VT-\delta_{I}I-\kappa_{N}IF-\kappa_{E}IE, \end{array}$$

(7) $$\begin{array}{cc} \frac{dR}{dt} & =\phi FT-\rho R, \end{array}$$

(8) $$\begin{array}{cc} \frac{dF}{dt} & =p_{F}I-\delta_{F}F, \end{array}$$

(9) $$\begin{array}{cc} \frac{dC_{n}}{dt} & =-\beta_{Cn}\left(\frac{V}{V+h_{C}}\right)C_{n}, \end{array}$$

(10) $$\begin{array}{cc} \frac{dE}{dt} & =\beta_{Cn}\left(\frac{V(t-\tau_{C})}{V\left(t-\tau_{C}\right)+h_{C}}\right)C_{n}\left(t-\tau_{C}\right)e^{p_{C}}-\delta_{E}E, \end{array}$$

(11) $$\begin{array}{cc} \frac{dB_{n}}{dt} & =-\beta_{Bn}\left(\frac{V}{V+h_{B}}\right)B_{n}, \end{array}$$

(12) $$\begin{array}{cc} \frac{dP}{dt} & =\beta_{Bn}\left(\frac{V(t-\tau_{B})}{V\left(t-\tau_{B}\right)+h_{B}}\right)B_{n}\left(t-\tau_{B}\right)e^{p_{B}}-\delta_{P}P, \end{array}$$

(13) $$\begin{array}{cc} \frac{dA_{S}}{dt} & =p_{S}P-\delta_{S}A_{S}, \end{array}$$

(14) $$\begin{array}{cc} \frac{dA_{L}}{dt} & =p_{L}P-\delta_{L}A_{L}. \end{array}$$

The model is a coupled system constituting three major parts. The first part (Equations (4)–(7)) describes the process of infection of target cells by influenza virus, retaining the skeleton of the TIV model except for the addition of several components related to the immune response. $\kappa_{S}VA_{S}$ and $\kappa_{L}VA_{L}$ represent virus neutralisation by antibodies (both a short-lived antibody response $A_{S}$ driven by, for example IgM, and a longer-lasting antibody response $A_{L}$ driven by, for example IgG and IgA [28,29]. The innate immune response, mediated by interferon (IFN; *F*), triggers target cells (*T*) to become virus-resistant (*R*) at a rate ϕF, and the resistant cells may lose protection and change back to target cells at a rate ρ [30]. Infected cells are killed by IFN-activated natural killer cells at a rate $\kappa_{N}F$ [30,31] and by effector ${\mathrm{CD8}}^{+}$ T cells (*E*) at a rate $\kappa_{E}E$ [29].

The second part (Equation (8)) describes the dynamics of IFN, the production of IFN by infected cells at rate $p_{F}$ and decay of IFN at rate $\delta_{F}$ [26,30]. Note that although we confine the innate immunity to be IFN-mediated in the model (because of the well-established role of IFN), the compartment IFN can also be viewed as a gross response of various innate immune processes to viral infection.

The last part (Equations (9)–(14)) describes various major adaptive immune responses including ${\mathrm{CD8}}^{+}$ T cells and B cell-produced antibodies. Naive ${\mathrm{CD8}}^{+}$ T cells ($C_{n}$), upon stimulation by antigen-presentation at a rate $\beta_{Cn}V/\left(V+h_{C}\right)$ (where $\beta_{Cn}$ is the maximum stimulation rate and $h_{C}$ indicates the viral load (*V*) at which half of the stimulation rate is achieved), initiate the proliferation/differentiation process and produce effector ${\mathrm{CD8}}^{+}$ T cells at a multiplication factor of $e^{p_{C}}$ after a delay $\tau_{C}$. The delay could be induced by both naive ${\mathrm{CD8}}^{+}$ T cell proliferation/differentiation and effector ${\mathrm{CD8}}^{+}$ T cell migration and localisation to the site of infection [32,33,34]. Similarly, naive B cells $B_{n}$ are recruited and subsequently produce antibody-secreting plasma B cells *P* at a rate $\beta_{Bn}V/\left(V+h_{B}\right)$ after a delay $\tau_{B}$. Two types of antibody responses, a short-lasting antibody response $A_{S}$ (e.g., IgM lasting from about day 5 to day 20 p.i.) and a longer-lived antibody response $A_{L}$ (e.g., IgG and IgA lasting weeks to months) [28,29], are modelled by simple linear production and decay kinetics. Effector ${\mathrm{CD8}}^{+}$ T cells and plasma B cells decay at rates $\delta_{E}$ and $\delta_{P}$ respectively.

As demonstrated above, the TIV model can be seen as a special case of the IR model where all immune components are knocked out. We use the same set of parameter values to simulate both models. For model simulation, the initial conditions are $\left(V,T,I\right)=\left(10^{4},T_{0},0\right)$ for the TIV model and $\left(V,T,I,F,R,C_{n},E,B_{n},P,A_{S},A_{L}\right)=\left(10^{4},T_{0},0,0,0,100,0,100,0,0,0\right)$ for the IR model. The parameter values are adopted from [27] where the IR model was fitted to a set of murine data from [29] and provided in Table 1. Note that we choose the parameter values such that the two models exhibit typical behaviours of the two model types observed in previous modelling studies (detailed in the summary section of the Results) and therefore are competent representatives of the model types.

### 2.3. The Pharmacokinetic Model

A one-compartment pharmacokinetic (PK) model with first-order drug absorption and first-order elimination is used to simulate the plasma OC concentration *D* [35]:

(15) $$\frac{dD}{dt}=\omega k_{a}D_{admin}e^{k_{a}\left(t-t_{admin}\right)}-k_{e}D,\;\left(t\geq t_{admin}\right),$$

where $D_{admin}$ (in units of mg) and $t_{admin}$ (in units of days) indicate the dose and time of oseltamivir administration respectively, $k_{a}$ is the rate of oseltamivir absorption into the plasma and $k_{e}$ is the rate of OC elimination. ω is a factor converting absorbed drug mass to OC concentration (in units of ng/mL). In this study, we shall concentrate on a standard regimen of 75 mg oseltamivir twice per day (note that this treatment is only recommended for patients aged 13 years and older). This means $D_{admin}=75\;\mathrm{mg}$ is applied every 12 h. We assume that the drug administration starts at 28 h p.i. in line with previous clinical and modelling studies [21,36]. $k_{a}=11.04\;{\mathrm{day}}^{-1}$ and $k_{e}=2.64\;{\mathrm{day}}^{-1}$ [21,37]. ω is chosen to be 4.63 ${\mathrm{kL}}^{-1}$ such that the simulated plasma OC concentration oscillates in a range consistent with the experimental estimate of 167–332 ng/mL (median minimum to median maximum) [37].

The PK model and the viral dynamic models are coupled through an OC concentration-dependent reduction in viral production. As introduced before, this is modelled by $\left(1-\epsilon \right)p_{V}$ in Equation (3) or Equation (4). ϵ is given by a function of OC concentration,

(16) $$\epsilon \left(t\right)=\frac{\epsilon_{max}D\left(t\right)}{D\left(t\right)+EC_{50}},$$

where $\epsilon_{max}$ represents the maximum antiviral effect achievable by oseltamivir and is chosen to be 0.98 according to [21]. Small perturbations in $\epsilon_{max}$ (or equivalently $p_{V}$) do not alter the model dynamics [27]. The concentration achieving half-maximal effect, $EC_{50}$, is highly variable (e.g., for different influenza strains or individual hosts) ranging from 0.0008–35μM [21] which is equivalent to approximately 0.2–9900 ng/mL (estimated using the OC molecular mass of 284.1736 g/mol). In this study, we only allow $EC_{50}$ to vary within this plausible range.

The PK-PD models are solved in MATLAB R2016b (The MathWorks, Natick, MA, USA). The models are solved iteratively in time, and for each time step MATLAB’s ODE solver *ode15s* is used to calculate the solutions. MATLAB code is provided in the Supplementary Material.

### 2.4. Infection-Related Statistics

**Figure 2:**
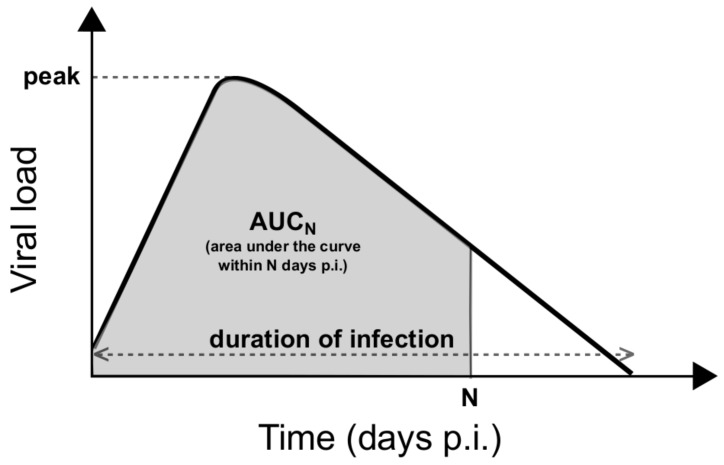
Schematic diagram showing the definitions of three important quantities characterising the viral load profile. Peak viral load indicates the maximum of the viral load curve. Duration of infection is the period of time from the start of infection to the time when the viral load reaches the limit of detection. The area under the viral load curve within the first *N* days p.i. (${\mathrm{AUC}}_{N}$; calculated by integrating the viral load over the relevant period) is a measure of the cumulative viral load over a certain period of infection.

For influenza, viral load is one of the most important indicators of the progress of infection. Therefore measurable quantities related to the viral load may be used to improve the overall assessment of the efficacy of antiviral therapies. In this paper, by varying $EC_{50}$ (as a way to vary drug efficacy), we investigate three important infection-related quantities: (1) the area under the viral load curve (AUC); (2) the peak viral load; and (3) the duration of infection. These statistics are schematically illustrated in Figure 2. Note that we redefine a truncated AUC within the first N days p.i. (${\mathrm{AUC}}_{N}$; calculated by integrating the viral load (before log-transform) over the relevant period), which generalises the traditionally-defined AUC. Note that, in the Results, the ${\mathrm{AUC}}_{N}$ and the peak viral load are normalised to their corresponding quantities in the no-drug control to aid comparison.

## 3. Results

In this section, we first compare the behaviour of the two virus dynamic models (in the absence of drug), in order to demonstrate the mechanistic difference in the control of viral growth and clearance. Then we investigate and compare the dependence of both viral load curve and infection-related quantities (such as the area under the curve, peak viral load, and duration of infection) on drug efficacy (implemented by varying $EC_{50}$) for both models. We also investigate and compare the predictions of the two models for varied drug administration times. Finally, we summarise the model predictions and evaluate them in relation to previous modelling and clinical studies.

### 3.1. Behaviour of the Viral Dynamic Models

**Figure 3:**
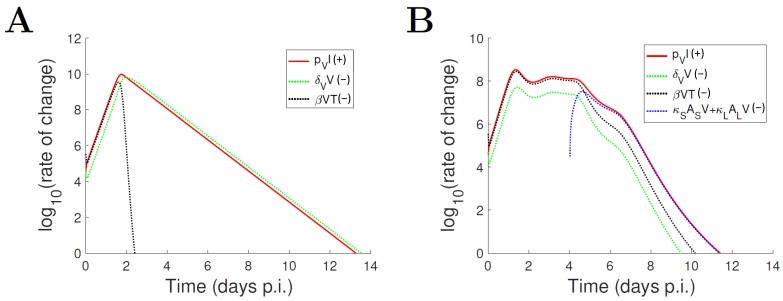
Results showing the contributions of various processes to the establishment of the viral load profile. (**A**) For the TIV model, three processes are involved: production of free virus ($p_{V}I$), unspecified virus clearance ($\delta_{V}V$) and binding to target cells (βVT). The terms appear on the righthand side of Equation (3); (**B**) For the IR model, four processes are involved: production of free virus ($p_{V}I$), unspecified virus clearance ($\delta_{V}V$), binding to target cells (βVT) and virus neutralisation by antibodies ($\kappa_{S}A_{S}V+\kappa_{L}A_{L}V$). The terms appear on the righthand side of Equation (4). The processes leading to an increase in viral load are labelled by (+) and shown by solid curves, while the processes reducing the viral load are labelled by (−) and shown by dotted curves.

We first demonstrate how the dynamical behaviour of the TIV model and the IR model differ due to the underlying mechanisms utilised to control virus. In the main text we focus on the viral load solution, which is the most direct indicator of the progress of infection and is easy to measure experimentally. Full model solutions are provided in Figures S1 and S2 in the Supplementary Material. Figure 3A shows the contribution of different processes, including production of free virus ($p_{V}I$, which appears on the righthand side of Equation (3)), unspecified virus clearance ($\delta_{V}V$) and binding to target cells (βVT), to the establishment of the viral load profile of the TIV model. We see that all the processes play a role in determining the rate of early viral growth (while depletion of target cells at approximately day 2 p.i., indicated by a rapid decrease of the term βVT (dotted black curve) leads to a subsequent dominant role for viral clearance). This is consistent with the well-established result that target cell-limited models exhibit a robust two phase response in the time course of viral load—an upstroke phase driven by exponential viral growth followed by a decrease phase of exponential viral clearance [38].

Similarly, for the IR model, we show the contribution of different processes including production of free virus ($p_{V}I$, which appears on the righthand side of Equation (4)), unspecified virus clearance ($\delta_{V}V$), binding to target cells (βVT) and virus neutralisation by antibodies ($\kappa_{S}A_{S}V+\kappa_{L}A_{L}V$) in Figure 3B. One of the notable features is that the level of target cells no longer limits the free virus binding (see the dotted black curve), demonstrating a very different mechanism for viral control compared to that of the TIV model. The innate immune response is activated within the first 2 days p.i. (Figure S2) and has been shown in [27] to suppress viral growth (which leads to the plateau in the red curve in Figure 3B). Following a lag phase due to B cell maturation (lasting approximately 4 days), antibodies quickly dominate viral clearance, particularly during the late stage of infection (Figure 3; dotted blue curve). The viral load profile for the IR model exhibits three phases (and is robust to parameter perturbations [27]), which is another distinguishing feature from the biphasic viral load in the TIV model.

### 3.2. The TIV Model Predicts a Relatively Complex Effect of Drug Efficacy on Viral Load Profile

**Figure 4:**
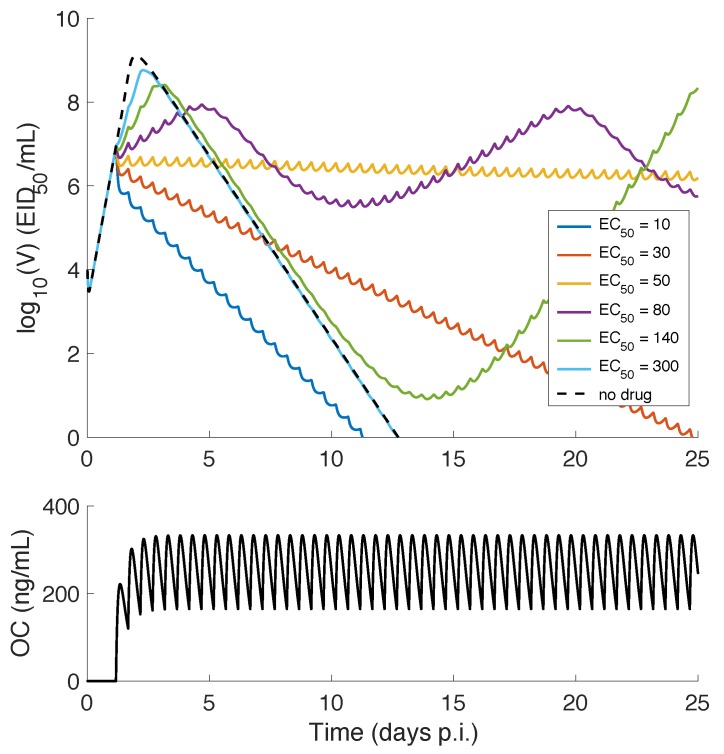
Dependence of viral load profile on drug efficacy for the TIV model. The time course of plasma oseltamivir carboxylate (OC) concentration is shown in (**B**). For $EC_{50}$ (half maximal effective concentration) varying from 10 ng/mL to 300 ng/mL, corresponding viral load solutions are shown in (**A**) with different colours. The solution with no drug applied is shown by the dashed black curve. ${\mathrm{EID}}_{50}/\mathrm{mL}$ : 50% egg infective dose.

When oseltamivir is first taken at 28 h p.i. (and subsequently taken every 12 h indefinitely), simulation based on the TIV model predicts that the viral load profile is highly dependent on drug efficacy (Figure 4; implemented by varying $EC_{50}$). For low $EC_{50}$ (i.e., high efficacy), for example 10 ng/mL, oseltamivir effectively reduces the viral load and accelerates the clearance of free virus (i.e., reaching the assumed detection of limit V=1 earlier than the no-drug control). When the $EC_{50}$ increases to 30 ng/mL, the reduction in viral load is accompanied by a significantly postponed clearance time. As the $EC_{50}$ is increased further to 50 ng/mL, an almost sustained elevation in viral load is achieved (in fact it is a very slow decay process in the long term). Further increases in $EC_{50}$, for example to 80 ng/mL or 140 ng/mL, leads to an oscillatory viral load, which is a result of the target cell replenishment due to the regrowth term in Equation (1) (Note that oscillations with large amplitudes may reach a very low level where virus may go extinct stochasticity before rebound, which has been studied in [26,39,40,41]). If target cell replenishment is inhibited (by setting $g_{T}=0$), the oscillations disappear (Figure S3 in the Supplementary Material). The above results are consistent with a recent finding that the inclusion of target cell regrowth can lead to chronic infection [42]. For a sufficiently high $EC_{50}$, e.g., 300 ng/mL, the viral load trajectory almost follows that of the no-drug control (dashed black curve), except for a slight reduction in peak viral load during the early phase of infection.

This dynamic profile for the viral load does not correlate well with drug efficacy for the TIV model. But we do identify a narrow window, of approximately two days immediately after drug administration, where the variation in $EC_{50}$ can be reliably indicated by the viral load level (Figure 4 and Figure S3). If the TIV model is an appropriate model for studying infection dynamics, this suggests that a more frequent measurement of viral load soon after drug administration may improve the estimation of drug efficacy. An important reason for this phenomenon is the availability of sufficient target cells in the early phase of infection. Once target cell depletion dominates viral clearance, little information about drug effect can be identified using the TIV model. In fact, as we will soon show, such a window is also applicable to the IR model, confirming the important role of the target cell pool in affecting model predictions.

### 3.3. Predicting the Dependence of Infection-Related Quantities on Drug Efficacy Using the TIV Model

**Figure 5:**
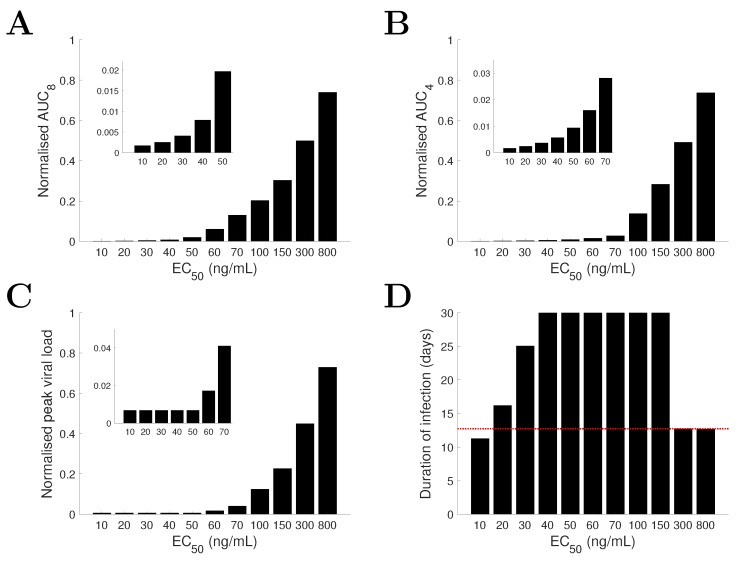
Dependence of infection-related quantities on drug efficacy for the TIV model. As drug efficacy $EC_{50}$ varies from 10 ng/mL to 800 ng/mL, infection-related quantities, under the curve (AUC) (**A**,**B**), peak viral load (**C**) and duration of infection (**D**), are shown in different panels. ${\mathrm{AUC}}_{8}$, ${\mathrm{AUC}}_{4}$ and peak viral load are normalised to their corresponding quantities in the no-drug control. Insets show sub-parts of the plots. For duration of infection longer than 30 days, we truncate the duration at 30 days in panel **D**. The duration of infection without antiviral treatment is indicated by the dotted red line in panel **D**.

Figure 5 shows how infection-related quantities vary with different $EC_{50}$ for the TIV model. As the $EC_{50}$ increases from 10 ng/mL to 800 ng/mL, ${\mathrm{AUC}}_{8}$ (the truncated AUC within the first 8 days p.i.; a time frame usually covers the symptomatic period of influenza infection for individual patients) also increases (Figure 5A). A truncated AUC within a shorter period of 4 days p.i., ${\mathrm{AUC}}_{4}$, is also examined and found to follow a similar trend to that of ${\mathrm{AUC}}_{8}$ (Figure 5B), suggesting that the trend in AUC statistics does not depend strongly on the choice of period (subject to the assumed initiation time of treatment). Peak viral load is also positively correlated with $EC_{50}$, suggesting a strong correlation with AUC. A similar correlation between peak viral load and AUC was previously identified by fitting a TIV model to clinical data for a placebo group [11], which is likely (at least partially) driven by the properties of the TIV model. However, we find that for very small $EC_{50}$ (i.e., very high drug efficacy), the peak viral load is insensitive to the change in $EC_{50}$ (see the inset of Figure 5C), which is caused by an immediate inhibition of viral growth upon drug administration such that the peak viral load is achieved almost at that time (see Figure 4).

When we consider the duration of infection, the relationship with $EC_{50}$ is more complicated (Figure 5D). For $EC_{50}<$ 30 ng/mL, increasing the $EC_{50}$ leads to an increase in the duration of infection. For a further increase in $EC_{50}$, e.g., to 40–150 ng/mL, the viral load never returns to the limit of detection (V=1) within a reasonable time frame (e.g., truncated at day 30 p.i. in Figure 5D) such that the measure of infection duration becomes undefined. Drug administration, at least for the TIV model, triggers a state of chronic infection. However, when the $EC_{50}$ is sufficiently large, e.g., 300 or 800 ng/mL, the duration of infection is insensitive to the change in $EC_{50}$ and is close to that of the no-drug control (dotted red line in Figure 5D), as the viral clearance curve almost follows the solution of the TIV model without treatment (dashed black curve in Figure 4).

In addition, given the fact that target cell regeneration may affect model predictions [42], we further perform simulations using the TIV model without target cell regrowth (i.e., $g_{T}=0$). Results show that target cell regrowth does not qualitatively alter the model predictions (Figure S4 in the Supplementary Material).

### 3.4. The IR Model Predicts a Relatively Robust Effect of Drug Efficacy on Viral Load Profile

**Figure 6:**
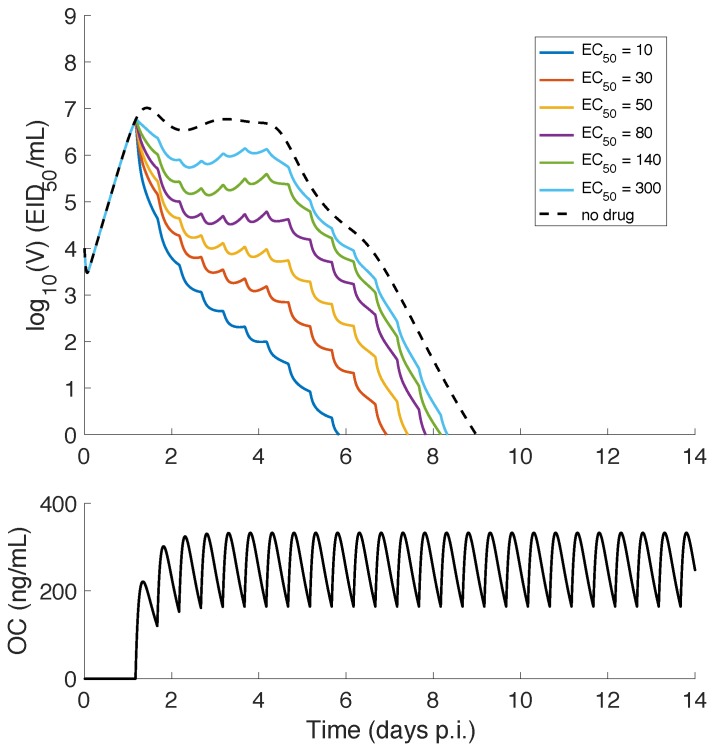
Dependence of viral load profile on drug efficacy for the IR model. The time course of plasma oseltamivir carboxylate (OC) concentration is shown in (**B**). For $EC_{50}$ varying from 10 ng/mL to 300 ng/mL, corresponding viral load solutions are shown in (**A**) with different colours. The solution with no drug applied is shown by the dashed black curve.

In contrast to the TIV model, the IR model does not support long-lasting infection dynamics for any $EC_{50}$ value. Drug administration always shortens the duration of infection and reduces the AUC (Figure 6). This is a direct result of the effective virus resolution by the immune response even in the absence of treatment. In detail, for a decreasing $EC_{50}$, the viral load also decreases over the entire infection period, suggesting that experimental measurement at any time during the infection would be useful in estimating drug efficacy. Hence, both the TIV model and the IR model predict that finely spaced viral load measurements during the period soon after drug administration should be informative for the evaluation of treatment efficacy. The IR model further predicts that measuring the viral load in a time window of approximately 3–5 days p.i. would be more sensitive to the change in drug efficacy (Figure 6).

### 3.5. Predicting the Dependence of Infection-Related Quantities on Drug Efficacy Using the IR Model

**Figure 7:**
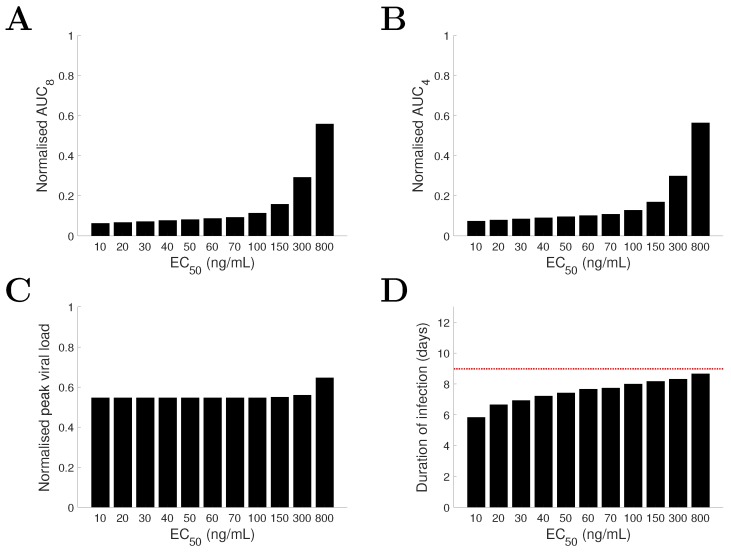
Dependence of infection-related quantities on drug efficacy for the IR model. As drug efficacy $EC_{50}$ varies from 10 ng/mL to 800 ng/mL, infection-related quantities, AUC (**A**,**B**), peak viral load (**C**) and duration of infection (**D**), are shown in different panels. ${\mathrm{AUC}}_{8}$, ${\mathrm{AUC}}_{4}$ and peak viral load are normalised to their corresponding quantities in the no-drug control. The duration of infection without antiviral treatment is indicated by the dotted red line in panel **D**.

As analysed above, the IR model predicts a positive correlation between the $EC_{50}$ and the overall level of viral load, which is validated by the monotonically increasing relationship between $EC_{50}$ and AUC (Figure 7A,B). A similar result was also predicted by the TIV model (Figure 5), suggesting that the use of AUC to indicate drug efficacy is robust to model selection. Again, the results for both ${\mathrm{AUC}}_{8}$ and ${\mathrm{AUC}}_{4}$ are consistent, demonstrating that the ability of AUC to indicate drug efficacy is largely unaffected by choice of the infection period.

However, contrary to the TIV model, the IR model predicts very different dependences of peak viral load and duration of infection on drug efficacy. The peak viral load is insensitive to the change in $EC_{50}$ over a relatively broad range (e.g., from 10–300 ng/mL) (Figure 7C), a result of immediate inhibition of viral growth by treatment (Figure 6). When the $EC_{50}$ is sufficiently large, e.g., 800 ng/mL, the peak viral load may show a detectable change (and a further increase in $EC_{50}$ will increase the peak viral load). The discrepancy in the $EC_{50}$-sensitive range for AUC and peak viral load suggests a weak correlation between AUC and peak viral load. The duration of infection is increased, as $EC_{50}$ increases, and approaches the duration of infection for the no-drug control (dotted red line in Figure 7D).

Similar to Figure S4, we also perform simulations using the IR model without target cell regrowth (i.e., $g_{T}=0$). Results show that target cell regrowth does not qualitatively alter the model predictions (Figure S5 in the Supplementary Material).

### 3.6. Model pRediction and Comparison for Varied Drug Administration Time

**Figure 8:**
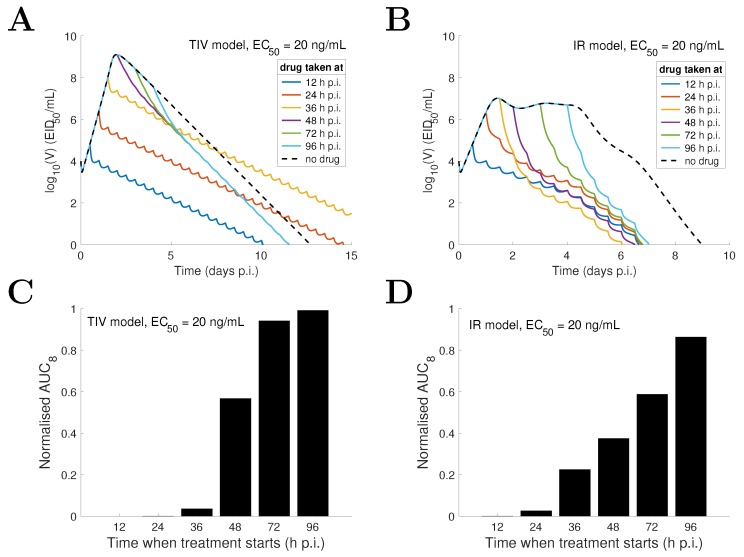
Comparison of model behaviours of the TIV model (**A**,**C**) and the IR model (**B**,**D**) for a highly effective drug ($EC_{50}=20$ ng/mL) and varied drug administration time. The time when drug is first taken is varied from 12 h p.i. to 96 h p.i. and the corresponding viral load solutions are shown in different colours. ${\mathrm{AUC}}_{8}$ is normalised to its corresponding quantity in the no-drug control. The solutions with no treatment are shown by dashed black curves.

In addition to varying drug efficacy (i.e., varying $EC_{50}$), varying the initiation time of drug administration may also significantly affect treatment outcomes [43,44,45]. Here we investigate the effect of drug administration time on the viral load profile for both the TIV model and the IR model. We look at two situations, one where the drug is highly efficient (e.g., $EC_{50}=$ 20 ng/mL), and the other where it is less efficient (e.g., $EC_{50}=$ 200 ng/mL). For $EC_{50}=$ 20 ng/mL, the TIV model predicts that early viral clearance, compared to the no-drug control (dashed black curve), can be achieved only when drug is first taken very early (e.g., 12 h p.i.) or relatively late (e.g., after 48 h p.i.) (Figure 8A). In contrast, the IR model predicts that early viral clearance can always be achieved regardless of when the treatment starts (Figure 8B). If we further assume that 12 h p.i. is the earliest time a patient may take drug (e.g., due to the latency from exposure to virus to the onset of symptoms), the TIV model predicts an optimal time of first drug administration of 12 h p.i. while the IR model predicts an optimal time of approximately 36 h p.i. (although the duration of infection is improved by less than one day compared to taking drug at other sub-optimal times; Figure 8B). In contrast to the duration of infection, the two models consistently show a decreasing AUC as the starting time of treatment is advanced (Figure 8C,D).

**Figure 9:**
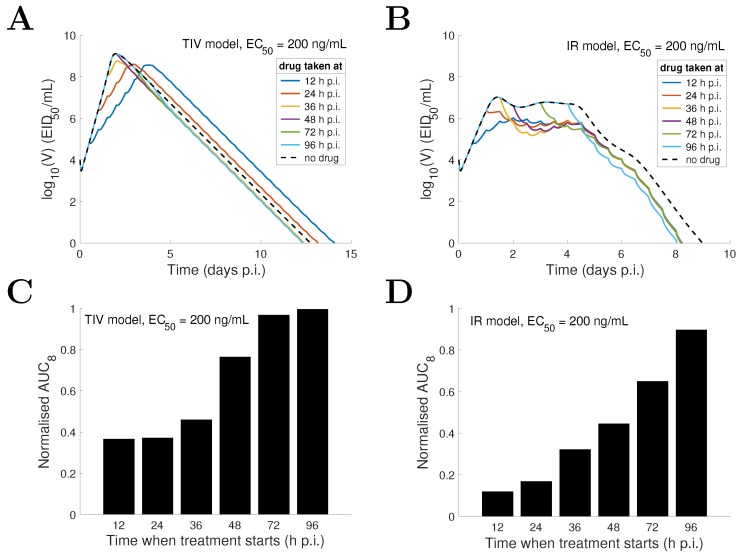
Comparison of model behaviours of the TIV model (**A**,**C**) and the IR model (**B**,**D**) for a less effective drug ($EC_{50}=200$ ng/mL) and varied drug administration time. The time when drug is first taken is varied from 12 h p.i. to 96 h p.i. and the corresponding viral load solutions are shown in different colours. ${\mathrm{AUC}}_{8}$ is normalised to its corresponding quantity in the no-drug control. The solutions with no treatment are shown by dashed black curves.

For $EC_{50}=$ 200 ng/mL, the TIV model predicts that the peak viral load is postponed and the duration of infection is prolonged when treatment starts earlier (Figure 9A). This is because reduced viral production delays the depletion of target cells and in turn delays the peak and clearance time of viral load. In contrast, the IR model predicts that varying the drug administration time does not significantly affect the duration of infection (Figure 9B; note that the duration of infection is always shortened whenever drug is first taken compared with that for the no-drug control). Despite this difference, both models again predict a positive correlation between the AUC and drug administration time (Figure 9C,D).

### 3.7. Results Summary

Here we summarise the model predictions and provide further comments on them based on previous studies:

- For a decreasing drug efficacy (indicated by an increasing $EC_{50}$), the TIV model predicts five types of behaviour (in terms of viral load kinetics and when compared with the no-drug control): (1) early clearance (see $EC_{50}$ = 10 ng/mL in Figure 4); (2) late clearance (see $EC_{50}$ = 30 ng/mL in Figure 4); (3) long-lasting infection (see $EC_{50}$ = 50 or 80 ng/mL in Figure 4); (4) an oscillatory viral load (see $EC_{50}$ = 80 or 140 ng/mL in Figure 4); and (5) an approach to the solution of the no-drug control (see $EC_{50}$ = 300 ng/mL in Figure 4). Note that without target cell regeneration, oscillatory-type chronic infection does not occur, while a delayed time to peak and clearance of free virus may be achieved (Figure S3 in the Supplementary Material). In contrast, the IR model predicts early viral clearance for all $EC_{50}$ and a shorter duration of infection when drug efficacy is higher (Figure 6). The different model behaviours have been observed in previous studies, for example Figure 2 in [17] and Figure 4 in [18] for the target cell-limited models and the middle panel of Figure 7 in [16] for the model incorporating a relatively detailed structure of the adaptive immune response. Therefore, we argue that the TIV model and the IR model used in our study are representative, and a detailed comparison of the model behaviours is important for the judgement of model predictions.
- Both models consistently predict that NAI efficacy is well correlated with the viral load level within a short time window (approximately 2 days) soon after drug administration (Figure 4 and Figure 6). This suggests that a high frequency measurement of viral load during that time window could improve the evaluation of in vivo drug efficacy. Although a very frequent measurement in clinical trials may not be practical, laboratory experiments using animals may be achievable.
- For the TIV model, the AUC and peak viral load are well correlated with NAI efficacy while the duration of infection varies in a different manner (Figure 5). For the IR model, the AUC and duration of infection are well correlated with NAI efficacy while the peak viral load is insensitive to the change in $EC_{50}$ (Figure 7). Exclusion of target cell regeneration does not qualitatively affect the model predictions (Figures S4 and S5). The prediction of the IR model is more consistent with previous clinical findings that treatment with NAI reduces both the AUC and duration of viral shedding [36,46]. On the other hand, the TIV model, which lacks an immune response, might be more appropriate to predict infection of immunocompromised/immunosuppressed patients where prolonged influenza viral shedding has been observed [47], although prolonged viral shedding was also observed in young immunocompetent adults [48]. We note that such a scenario is also highly related to the emergence of antiviral resistance [49].
- For a highly effective NAI (e.g., $EC_{50}=20$ ng/mL), the TIV model predicts that sufficient early drug administration may shorten the duration of infection, while the IR model predicts an optimal drug administration time of approximately 36 h p.i. when assessed by clearance time (Figure 8A,B). In contrast, for a less effective NAI (e.g., $EC_{50}=200$ ng/mL), the TIV model predicts that early drug administration may delay the peak in viral load and in turn prolong the duration of infection, while the IR model predicts that the duration of infection is insensitive to a change in drug administration time (Figure 9A,B). Note that we have only considered infection with and treatment of a drug-sensitive strain. We have not considered the possible adverse consequences of drug resistance. Early treatment within the first two days post-infection may increase the risk of emergence of drug-resistant strains [20], which we expect would lead to additional complexities in system behaviour.
- Clinical studies have found that early treatment is associated with reduced viral load and a shortened duration of viral shedding [48,50]. Thus, to qualitatively reproduce this finding, the TIV model requires that the NAI is highly effective and drug is first administrated before the peak viral load (see Figure 8A) while the IR model requires that the NAI is highly effective and drug is first administrated after the peak viral load (see Figure 8B). Interestingly, both models predict that a high NAI efficacy is necessary. However, since the onset of illness (and possibly the time to seek antiviral treatment) is approximately at the time of peak viral load [51], the IR model simulates a more realistic scenario and is expected to provide more reliable predictions. In addition, despite the different behaviours for the TIV model and the IR model, they both predict that earlier drug administration is associated with a lower AUC (Figure 8 and Figure 9).

## 4. Discussion

Models of within-host influenza viral dynamics have played an important role in advancing our understanding of viral kinetics and host-virus interactions over the past decade. Although numerous models have been proposed, most of them can be classified into two types in terms of the mechanism of viral control: the target cell-limited models and the immune response models. The target cell-limited models utilise depletion of target cells to limit the formation of infected cells and in turn the production of virus, while the immune response models rely on timely activation of innate and adaptive immune responses to clear infected cells and free virus. In this paper, we used two models, one with each of the alternative mechanisms for within-host influenza viral control, to compare the outcome of antiviral treatment (focusing on NAIs). Through varying drug efficacy, we found that the two models predict very different viral load profiles, which can be further quantified by infection-related quantities such as the AUC, peak viral load and duration of infection. In particular, the negative correlation between AUC and drug efficacy is robust to model selection. Our results demonstrate that the assumed mechanism of viral control (either target cell limitation or immune-mediated control) is a major driver of the observed differences in viral kinetics under antiviral treatment. However, clearly other model assumptions, such as the presence or absence of target cell regrowth, and the form of that regrowth if present, lead to a more general model-dependency in the predictions. We only claim that the mechanism of viral control is influential, but not the sole factor affected the predicted antiviral effect. With some support from previous clinical studies (as discussed in the last part of the Results), we conclude that the target cell-limited model is more suitable for modelling in vitro assays or infection in some immunocompromised/immunosuppressed patients where prolonged influenza viral shedding is evident, while the immune response model is preferred for predicting the infection/antiviral effect in immunocompetent animals/patients.

Viral shedding and associated transmission among individuals is highly heterogeneous [52,53,54]. Vegvari et al. found, by fitting a TIV model to a placebo group (9 patients) in a clinical trial, that peak viral load and AUC exhibit the greatest variation among individuals [11]. Large variation in those quantities may hinder their use as a baseline to reliably evaluate the efficacy of antiviral treatment, although our results suggest that AUC is a model-independent quantity to reflect the variation in drug efficacy. Vegvari et al. also found that the duration of infection was highly correlated with the basic reproduction number and was the most sensitive quantity to assess antiviral therapies in clinical trials. The IR model supports a reliable use of duration of infection to reflect drug efficacy (Figure 7) but reliability may become an issue for (even partially) immunocompromised/immunosuppressed patients whose infection and treatment outcomes may be better predicted by the TIV model (Figure 2). To resolve these problems, we recommend both testing the uncertainty of AUC for a larger number of placebo patients and examining the patients’ immune responses before choosing the duration of infection (in terms of viral load) as a reliable virological endpoint.

Influenza pathogenesis is associated with both a high viral load and hyper-cytokinemia [55]. Thus, besides the virological endpoints, quantities related to the immune response, such as the levels of cytokine and chemokine expression, may also reflect the severity of infection [56,57,58] and therefore are plausible candidates to facilitate the assessment of antiviral therapies. Introducing immune factors to quantify the severity of infection in mathematical models has been proposed in previous studies [59,60] and is a promising and cost-effective way to predict the effect of antiviral treatment on the expression of immune responses. By enriching available viral dynamic models with more detailed immune components, we plan to establish a comprehensive and reliable model to assist in the design and assessment of antiviral treatment regimens in the near future.

An important application of viral dynamic models is the estimation of key kinetic parameter through model fitting, which is, however, strongly affected by the issue of parameter identifiability [61]. The TIV model has been widely used to estimate the viral replication rate and viral clearance rate because of its simple structure and small number of parameters. But the only data available for the TIV model is the measurement of viral load at discrete time points, which has been shown to be insufficient to unambiguously determine all kinetic parameters [10,62]. An even more challenging problem is expected for models with various immune components which have a large number of parameters. Although some immune response data is available (note that measurement of the in vivo immune response usually requires animal sacrifice and is costly), it is usually insufficient to overcome the dramatic expansion in parameter space dimensionality [25,27,29]. Due to non-identifiability issues and other factors such as variation among different individuals/species/assays, kinetic parameter values estimated in different studies can differ substantially and most model-based assessments of antiviral effects including ours have been focused on qualitative predictions. Hence, development of a more comprehensive and realistic model of in vivo viral dynamics and methods to reduce the uncertainty in parameter estimation are required in order to improve quantitative predictions of both host-virus interactions and antiviral effects.

## Figures and Tables

**Table 1:** Model parameter values for both the target cells—virus-infected cells—free virus (TIV) model and the immune response (IR) model. The values are adopted from one of our earlier study [27] where some of the parameters were obtained from the literature (references provided in [27]) and the rest were obtained by fitting the IR model to experimental data from [29]. $\left[u_{V}\right]$, $\left[u_{F}\right]$ and $\left[u_{A}\right]$ represent the units of viral load, interferon (IFN) and antibodies respectively. $\left[u_{V}\right]$ and $\left[u_{A}\right]$ are ${\mathrm{EID}}_{50}/\mathrm{mL}$ (50% egg infective dose) and pg/mL. IFN is assumed to be a non-dimensionalised variable in the model, and therefore $\left[u_{F}\right]$ can be ignored. URT: upper respiratory tract.

Par.	Description	Value & Unit
$T_{0}$	initial/total number of epithelial cells in the URT	$7\times 10^{7}$ cells
$g_{T}$	basal growth rate of healthy cells	$0.8\;d^{-1}$
$p_{V}$	viral production rate	210$\left[u_{V}\right]$${\mathrm{cell}}^{-1}d^{-1}$
$p_{F}$	IFN production rate	$10^{-5}\;$$\left[u_{F}\right]$${\mathrm{cell}}^{-1}d^{-1}$
$p_{S}$	short-lived antibody production rate	$12\;\left[u_{A}\right]{\mathrm{cell}}^{-1}d^{-1}$
$p_{L}$	long-lived antibody production rate	$4\;\left[u_{A}\right]{\mathrm{cell}}^{-1}d^{-1}$
$\delta_{V}$	nonspecific viral clearance rate	5 $d^{-1}$
$\delta_{I}$	nonspecific death rate of infected cells	2 $d^{-1}$
$\delta_{F}$	IFN degradation rate	2 $d^{-1}$
$\delta_{E}$	death rate of effector ${\mathrm{CD8}}^{+}$ T cells	0.57 $d^{-1}$
$\delta_{P}$	death rate of plasma B cells	0.5 $d^{-1}$
$\delta_{S}$	short-lived antibody consumption rate	2 $d^{-1}$
$\delta_{L}$	long-lived antibody consumption rate	0.015 $d^{-1}$
β	rate of viral consumption by binding to target cells	$5\times 10^{-7}\;{\mathrm{cell}}^{-1}d^{-1}$
$\beta^{\prime }$	rate of infection of target cells by virus	$3\times 10^{-8}\;{\left[u_{V}\right]}^{-1}d^{-1}$
ϕ	rate of conversion to virus-resistant state	0.33 ${\left[u_{F}\right]}^{-1}d^{-1}$
ρ	rate of recovery from virus-resistant state	2.6 $d^{-1}$
$\kappa_{S}$	rate of viral neutralization by short-lived antibodies	0.8 ${\left[u_{A}\right]}^{-1}d^{-1}$
$\kappa_{L}$	rate of viral neutralization by long-lived antibodies	0.4 ${\left[u_{A}\right]}^{-1}d^{-1}$
$\kappa_{N}$	killing rate of infected cells by IFN-activated NK cells	2.5 ${\left[u_{F}\right]}^{-1}d^{-1}$
$\kappa_{E}$	killing rate of infected cells by effector ${\mathrm{CD8}}^{+}$ T cells	$5\times 10^{-5}\;{\mathrm{cell}}^{-1}d^{-1}$
$\beta_{Cn}$	maximum rate of stimulation of naive ${\mathrm{CD8}}^{+}$ T cells by virus	1 $d^{-1}$
$\beta_{Bn}$	maximum rate of stimulation of naive B cells by virus	0.03 $d^{-1}$
$h_{C}$	half-maximal stimulating viral load for naive ${\mathrm{CD8}}^{+}$ T cells	$10^{4}\;\left[u_{V}\right]$
$h_{B}$	half-maximal stimulating viral load for naive B cells	$10^{4}\;\left[u_{V}\right]$
$p_{C}$	exponent of the $\mathrm{CD}8^{+}$ T cell multiplication factor	$7.2\;d^{-1}$
$p_{B}$	exponent of the B cell multiplication factor	$2.08\;d^{-1}$
$\tau_{C}$	delay for effector ${\mathrm{CD8}}^{+}$ T cell production	6 d
$\tau_{B}$	delay for plasma B cell production	4 d

## Supplementary Materials

The following are available online at www.mdpi.com/1999-4915/9/8/197/s1.
