# Supplementary material for: The Mechanisms for Within-Host Influenza Virus Control Affect Model-Based Assessment and Prediction of Antiviral Treatment
Source: Viruses. 2017 Jul 26;9(8):197. doi: 10.3390/v9080197 (PMC5580454; doi:10.3390/v9080197)
Supplement: Supplementary file 1 [file viruses-09-00197-s001.pdf]

Supplementary material for "The mechanisms for within-host influenza virus control affect model-based assessment and prediction of antiviral treatment"

Pengxing Cao[1] and James M. McCaw*[1,2,3]

[1]School of Mathematics and Statistics, The University of Melbourne, Melbourne, Victoria, Australia.
[2]Centre for Epidemiology and Biostatistics, Melbourne School of Population and Global Health, The University of Melbourne, Melbourne, Victoria, Australia.
[3]Modelling and Simulation, Infection and Immunity Theme, Murdoch Childrens Research Institute, The Royal Children's Hospital, Parkville, Victoria, Australia.

*Correspondence: jamesm@unimelb.edu.au

Contents

The supplementary material contains the following:

1. Supplementary figures (S1–S5)
2. MATLAB code for solving the TIV model
3. MATLAB code for solving the IR model

1 Supplementary figures

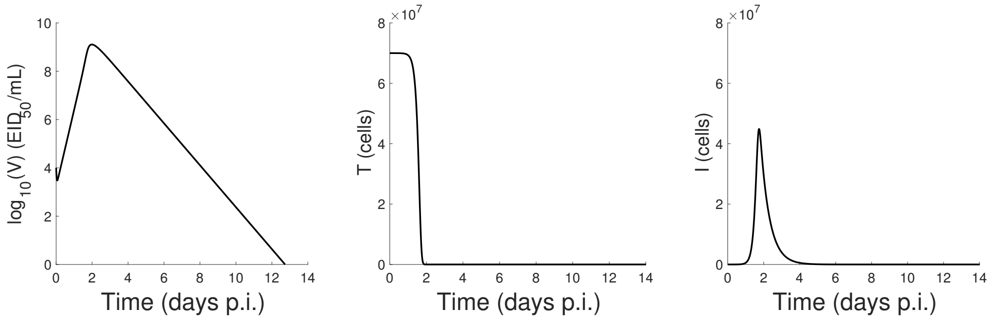

FIGURE S1: A solution of the TIV model with parameters given in Table S1 (no treatment).

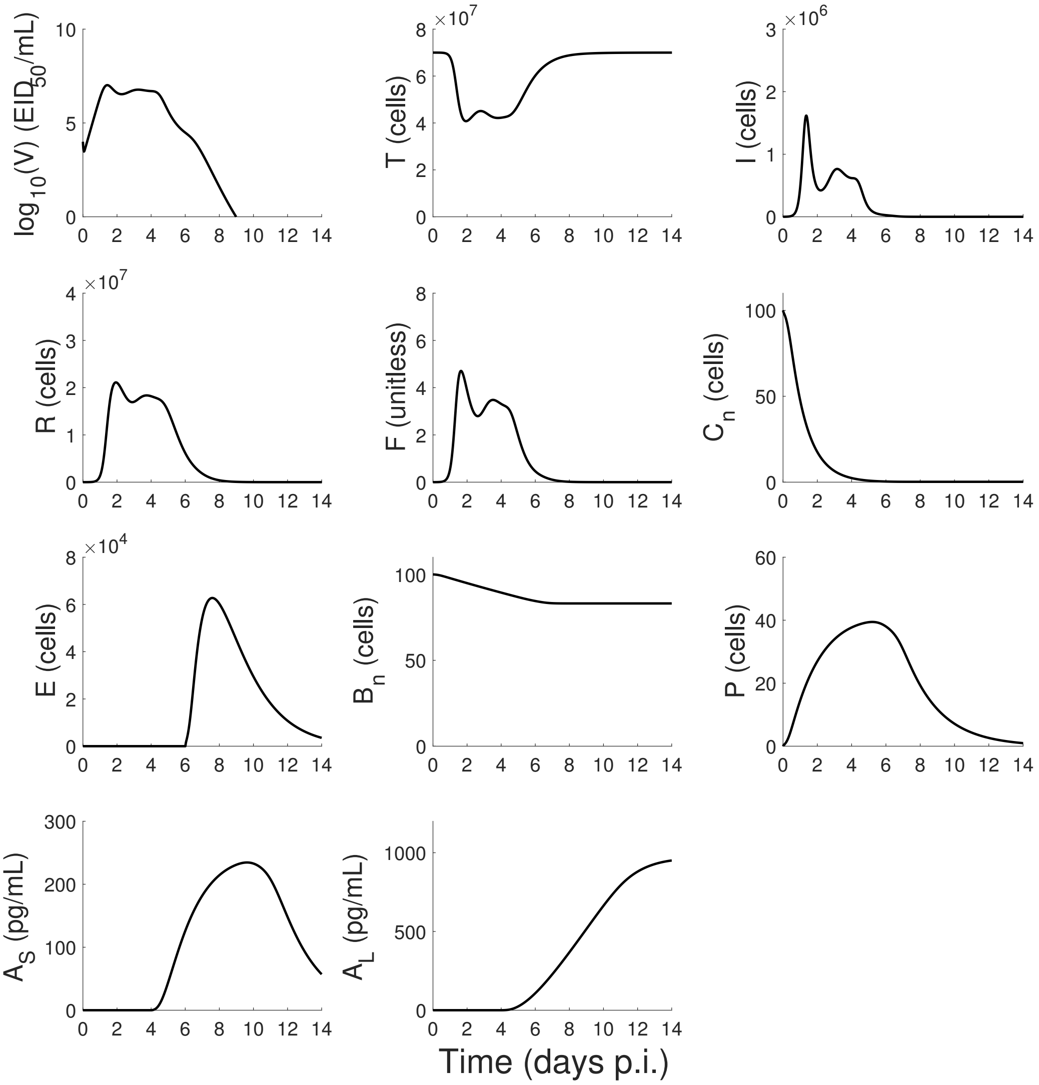

FIGURE S2: A solution of the IR model with parameters given in Table S1 (no treatment).

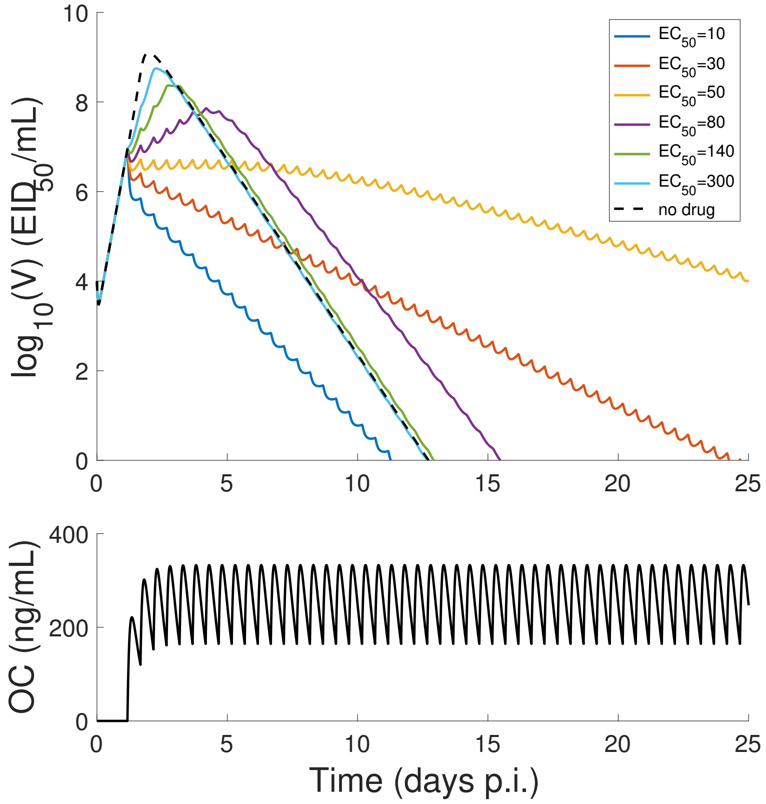

Figure S3: Dependence of viral load profile on drug efficacy for the TIV model. The time course of plasma OC concentration is shown in the lower panel. For EC_{50} varying from 10 ng/mL to 300 ng/mL, corresponding viral load solutions are shown in the upper panel with different colours. The solution with no drug applied is shown in dashed black curve. In this simulation, target cell regrowth is not allowed by setting $g_T = 0$.

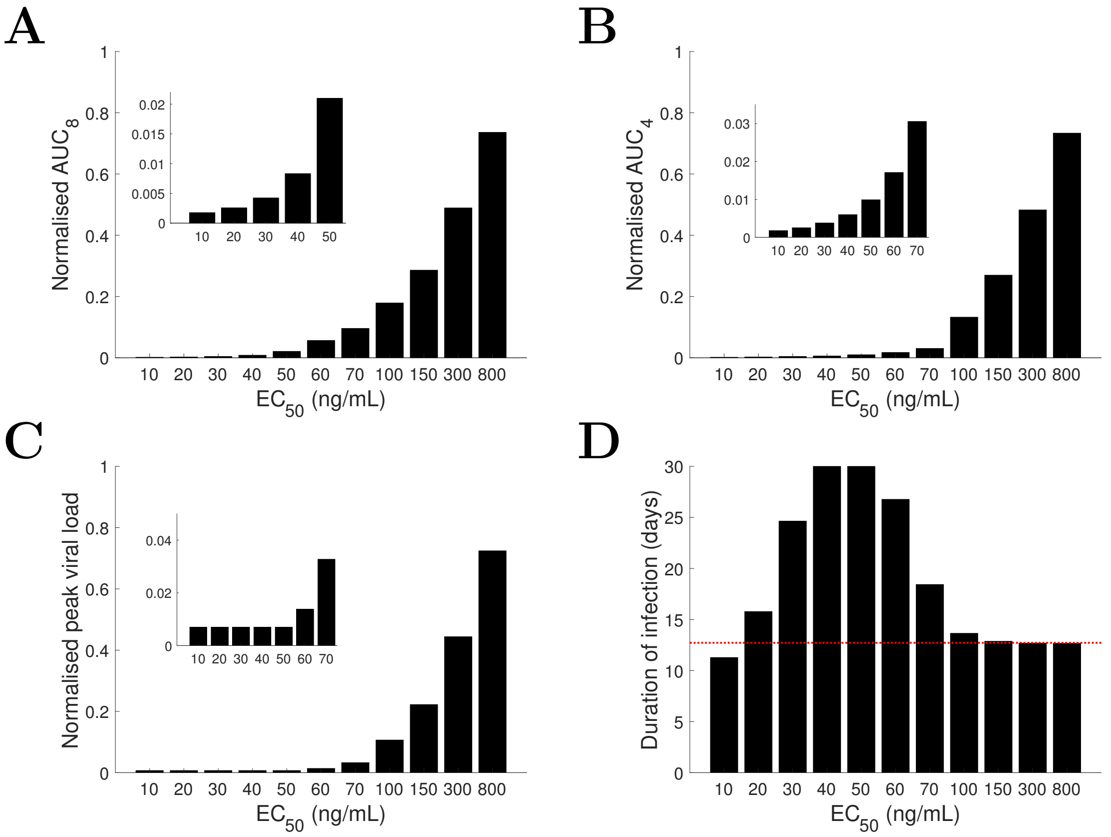

Figure S4: Dependence of infection-related quantities on drug efficacy for the TIV model with no target cell regrowth. As drug efficacy EC_{50} varies from 10 ng/mL to 800 ng/mL, infection-related quantities, AUC (**A** and **B**), peak viral load (**C**) and duration of infection (**D**), are shown in different panels. AUC_8, AUC_4 and peak viral load are normalised to their corresponding quantities in the no-drug control. Insets show sub-parts of the plots. For duration of infection longer than 30 days, we truncate the duration at 30 days in panel **D**. The duration of infection without antiviral treatment is indicated by the dotted red line in panel **D**.

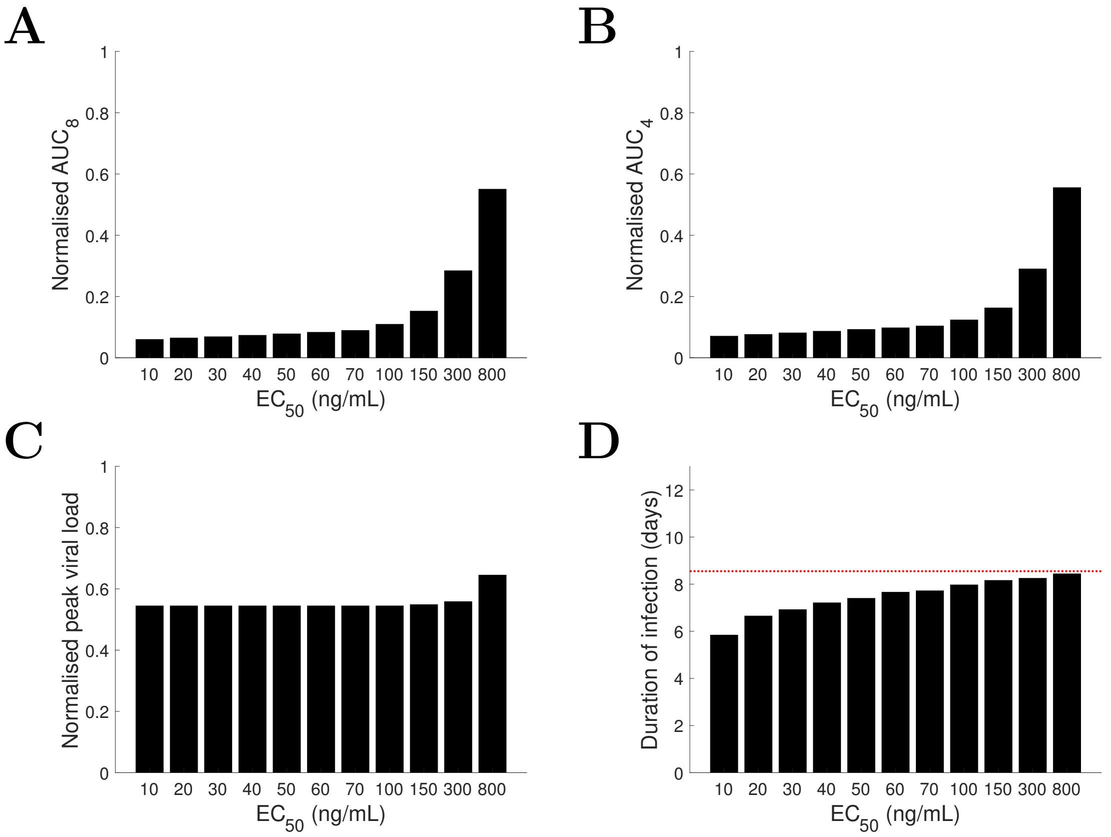

Figure S5: Dependence of infection-related quantities on drug efficacy for the IR model with no target cell regrowth. As drug efficacy EC_{50} varies from 10 ng/mL to 800 ng/mL, infection-related quantities, AUC (**A** and **B**), peak viral load (**C**) and duration of infection (**D**), are shown in different panels. AUC_8, AUC_4 and peak viral load are normalised to their corresponding quantities in the no-drug control. The duration of infection without antiviral treatment is indicated by the dotted red line in panel **D**.

2 MATLAB code for solving the TIV model

```
dt=0.01; % time step size
time=0:dt:20; % simulation time

% viral dynamic model parameters
pV=210;
beta=5e-7;
betap=3e-8;
V0=1e+4;
T0=7e+7;
gT=0.8;
deltaV=5;
deltaI=2;

% PK parameters
ka=11.04; % per day
ke=2.64; % per day
EC50=30; % ng/mL (varied)
emax=0.98; % epsilon_max
omega=4.63; % a factor converting mg to ng/mL

% variable vectors and initial conditions
V=zeros(1,length(time));
V(1)=V0;T=V;T(1)=7e+7;I=T;I(1)=0;De=I;D=I;

Dadmin=75; % applied NAI dose every 12 hours
% set Dadmin = 0 for no-drug case

Td0=28/24; % start of treatment (day)
% vary Td0 to change the drug administration time
Td=12/24; % period of dosage (one dose per 12 hours)
Td_ind=round((Td0:Td:time(end))./dt+1);

Tadmin=zeros(size(De));
Tadmin(Td_ind)=Dadmin;

init=[V(1),T(1),I(1),De(1),D(1)]'; % initial condition

options = odeset('RelTol',1e-3,'AbsTol',1e-6);

for i=2:length(time)
    [~,Y] = ode15s(@TIVmodel,[0 dt],init,options,gT,pV,beta,
        betap,deltaV,deltaI,T0,ka,ke,EC50,emax,omega);
    V(i)=Y(end,1);T(i)=Y(end,2);I(i)=Y(end,3);
    De(i)=Y(end,4)+Tadmin(i);
    D(i)=Y(end,5);
    % initial condition for next iteration
```

```
    init=[V(i),T(i),I(i),De(i),D(i)]';
end
```

where the function “TIVmodel” appearing in the command of *ode15s* is given by

```
function ynew=TIVmodel(~,y,gT,pV,beta,betap,deltaV,deltaI,T0,
    ka,ke,EC50,emax,omega)

ynew=zeros(5,1);

%viral dynamic model
ynew(1)=(1-emax*y(5)/(y(5)+EC50))*pV*y(3)-deltaV*y(1)-beta*y
    (1)*y(2); % equation of dV/dt
ynew(2)=gT*y(2)*(1-(y(2)+y(3))/T0)-betap*y(1)*y(2); % equation
    of dT/dt
ynew(3)=betap*y(1)*y(2)-deltaI*y(3); % equation of dI/dt

%PK model
ynew(4)=-ka*y(4);
ynew(5)=omega*ka*y(4)-ke*y(5); % equation of dD/dt
```

3 MATLAB code for solving the IR model

```
dt=0.01; % time step size
time=0:dt:20;

% PD parameters
pV=210;beta=5e-7;betap=3e-8;kappaE=5e-5;pF=1e-5;kappaS=0.8;
kappaL=0.4;betaBn=0.03;deltaP=0.5;deltaS=2; pS=12;pL=4;
deltaL=0.015;V0=1e+4;hC=1e+4; hB=1e+4;T0=7e+7;gT=0.8;
deltaV=5;deltaI=2;kappaN=2.5;pB=20.8;pC=7.2;phi=0.33;
rho=2.6;deltaF=2;betaCn=1;deltaE=0.57;tauC=6;tauB=4;

% PK parameters
ka=11.04; % per day
ke=2.64; % per day
EC50=30; % ng/mL (varied)
emax=0.98; % epsilon_max
omega=4.63; % a factor converting mg to ng/mL

% index indicating when delayed process starts
indC=round(tauC/dt+1); % for tauC
indB=round(tauB/dt+1); % for tauB

% variable vectors and initial conditions
V=zeros(1,length(time));
V(1)=V0;T=V;T(1)=7e+7;
I=T;I(1)=0;R=I;F=I;
Cn=100*ones(1,length(time));
Bn=100*ones(1,length(time));
E=zeros(1,indC+length(time));
P=I;AS=zeros(1,indB+length(time));
AL=AS;De=I;D=I;

Dadmin=75; % applied NAI dose every 12 hours
% set Dadmin = 0 for no-drug case

Td0=28/24; % start of treatment (day)
% vary Td0 to change the drug administration time
Td=12/24; % period of dosage (one dose per 12 hours)
Td_ind=round((Td0:Td:time(end))./dt+1);

Tadmin=zeros(size(De));
Tadmin(Td_ind)=Dadmin;

init=[V(1),T(1),I(1),R(1),F(1),Cn(1),E(1),Bn(1),P(1),AS(1),AL(1),De(1),D(1)]'; % initial condition

options = odeset('RelTol',1e-3,'AbsTol',1e-6);
```

```
for i=2:length(time)
    [~,Y] = ode15s(@IRmodel,[0 dt],init,options,E(i),AL(i),AS(i),phi,rho,deltaF,gT,pF,pV,beta,betap,kappaN,deltaV,deltaI,betaCn,betaBn,kappaE,kappaS,pL,pS,deltaL,deltaS,deltaP,deltaE,pC,pB,kappaL,hC,hB,T0,ka,ke,EC50,emax,omega);
    V(i)=Y(end,1);T(i)=Y(end,2);I(i)=Y(end,3);R(i)=Y(end,4);
    F(i)=Y(end,5);Cn(i)=Y(end,6);Bn(i)=Y(end,8);P(i)=Y(end,9);
    E(indC+i)=Y(end,7);
    AS(indB+i)=Y(end,10);
    AL(indB+i)=Y(end,11);
    De(i)=Y(end,12)+Tadmin(i);
    D(i)=Y(end,13);
    % initial condition for next iteration
    init=[V(i),T(i),I(i),R(i),F(i),Cn(i),E(indC+i),Bn(i),P(i),AS(indB+i),AL(indB+i),De(i),D(i)]';
end
```

where the function “IRmodel” appearing in the command of *ode15s* is given by

```
function ynew=IRmodel(~,y,E,AL,AS,phi,rho,deltaF,gT,pF,pV,beta,betap,kappaN,deltaV,deltaI,betaCn,betaBn,kappaE,kappaS,pL,pS,deltaL,deltaS,deltaP,deltaE,pC,pB,kappaL,hC,hB,T0,ka,ke,EC50,emax,omega)

ynew=zeros(13,1);
%viral dynamic model
ynew(1)=(1-emax*y(13)/(y(13)+EC50))*pV*y(3)-deltaV*y(1)-kappaS*y(1)*AS-kappaL*y(1)*AL-beta*y(1)*y(2); % equation of dV/dt
ynew(2)=gT*(y(2)+y(4))*(1-(y(2)+y(3)+y(4))/T0)-betap*y(1)*y(2)+(rho*y(4)-phi*y(2)*y(5)); % equation of dT/dt
ynew(3)=betap*y(1)*y(2)-deltaI*y(3)-kappaN*y(3)*y(5)-kappaE*y(3)*E; % equation of dI/dt
ynew(4)=(phi*y(2)*y(5)-rho*y(4)); % equation of dR/dt
ynew(5)=pF*y(3)-deltaF*y(5); % equation of dF/dt
ynew(6)=-betaCn*y(1)./(y(1)+hC)*y(6); % equation of dC_n/dt
ynew(7)=betaCn*y(1)./(y(1)+hC)*y(6)*exp(pC)-deltaE*y(7); % equation of dE/dt
ynew(8)=-betaBn*y(1)./(y(1)+hB)*y(8); % equation of dB_n/dt
ynew(9)=betaBn*y(1)./(y(1)+hB)*y(8)*exp(pB)-deltaP*y(9); % equation of dP/dt
ynew(10)=pS*y(9)-deltaS*y(10); % equation of dA_S/dt
ynew(11)=pL*y(9)-deltaL*y(11); % equation of dA_L/dt

%PK model
ynew(12)=-ka*y(12);
ynew(13)=omega*ka*y(12)-ke*y(13); % equation of dD/dt
```
